# Millennium development Goal 5: progress and challenges in reducing maternal deaths in Ghana

**DOI:** 10.1186/s12884-016-0840-0

**Published:** 2016-03-09

**Authors:** Minerva Kyei-Nimakoh, Mary Carolan-Olah, Terence V. McCann

**Affiliations:** Centre for Chronic Disease, College of Health and Biomedicine, Victoria University, PO Box 14428, Melbourne, VIC 8001 Australia

**Keywords:** Millennium development goals, MDG 5, Ghana, Obstetric care, Maternal deaths, Maternal health, Maternal mortality ratio

## Abstract

**Background:**

High maternal deaths in developing countries are recognised as a public health issue. To address this concern, targets were set as part of the Millennium Development Goals, launched in 2000 by the United Nations General Assembly. However, despite focused efforts, the maternal health targets in developing regions may not be achieved by 2015.

**Discussion:**

We highlight progress and challenges in reducing maternal deaths, with a particular focus on Ghana. We discuss key issues like the free maternal healthcare package, transportation and referral concerns, human resources challenges, as well as the introduction of direct-entry midwifery training and the Community-based Health and Planning Services rolled out to specifically help curb poor maternal health outcomes.

**Summary:**

A key contribution to the country’s slow progress towards achieving Millennium Development Goal 5 is that policy choices have often been in response to emergency or advancing problems rather than the use of preventive measures. Ghana can benefit greatly from long-term preventive strategies, the development of human resources, infrastructure and community health education.

## Background

In the late 20th century, maternal health gained international attention as a public health issue [1]. The increased interest was driven partly by the ‘Safe Motherhood Initiative’, launched in 1987, at the International Conference on Safe Motherhood, in Nairobi. In 1994, women’s right to safe pregnancy and childbirth were recognised at the United Nation’s International Conference on Population and Development, and adopted formally within its programme of action [2]. The Safe Motherhood Initiative included a goal to reduce maternal morbidity and mortality by half, by 2000 [3]. Though the initial aims of this initiative were not achieved by 2000, it contributed greatly in setting the stage for the introduction of the more rigorous efforts and policies seen today.

Since 2000, global attention has turned to the Millennium Development Goals (MDGs), which were developed after the Millennium Summit in 2000, and involved 189 nations as initial signatories, including Ghana. The MDGs were established by governments around the world under the United Nations Millennium Declaration. The central focus of the MDGs was to address socio-economic and health-related inequities [4], in areas such as poverty, education, gender equality, child mortality, maternal health, and infectious diseases, by the end of 2015 [5]. Eight broad development goals, along with measurable outcomes, were outlined, offering a path for directing efforts and a framework for tracking progress.

Previous international collaboration did not yield expected rewards in women’s reproductive health issues, particularly, in developing countries. In response, the fifth of the eight MDGs was included specifically to advance maternal health. This particular goal aimed to reduce maternal mortality ratio (MMR) by 75 % by 2015 (Target 5A) and to achieve universal access to reproductive health (Target 5B) [5]. MDG 5 is important because it formed the foundation for reducing maternal deaths in Sub-Saharan Africa. Between 1990 and 2013, global MMR decreased by 45 %; however, the targets for MDG 5 were not realised by many Sub-Saharan African and Southern Asian countries [6], where MMR continues to be very high.

## Maternal deaths in Africa

Global MMR for 2013 was 210 per 100,000 live births. In the same year, Sub-Saharan Africa’s MMR was 510 per 100,000 live births compared to a developing region like Eastern Asia, which had the lowest rate at 33 per 100,000 live births [6]. Consequently, in 2013, the lifetime risk of maternal death for women in Sub-Saharan Africa was 1 in 38, compared to 1 in 1,800 for women in Eastern Asia and 1 in 3,700 in developed regions [6]. Rural women in Sub-Saharan Africa have the least access to quality health service delivery, at the same time as having the highest MMR. Although conditions have improved considerably since 1990, a rural–urban gap has persisted. The proportion of births attended by skilled personnel, crucial for reducing perinatal, neonatal and maternal deaths, remains low [6].

In the African Region, Equatorial Guinea, Eritrea, and Rwanda had achieved the goals of MDG 5 by 2013. Some countries (Kenya, Guinea-Bissau and Central African Republic), made insufficient progress while other countries even saw a rise (South Africa, Somalia and Zimbabwe), in maternal deaths. Many others (Ghana, Ethiopia and Senegal) experienced a slow decline [6]. As noted by the United Nations Population Fund [7], recent declines in global MMR, particularly evident in the achievements of countries like Equatorial Guinea, Eritrea, Egypt and Bolivia, confirms that with commitment and appropriate resource allocation, strategies for reducing maternal deaths can produce excellent results, even in resource-poor settings. Therefore, considering that 2015 has ended, it is imperative to not only strengthen efforts but also understand why past interventions have not yielded anticipated outcomes. Several nations are currently evaluating their successes or shortcomings regarding the MDGs. This paper contributes to the discussion of maternal deaths with particular focus on evaluating Ghana’s progress and challenges in reducing MMR.

## Ghana

Ghana, a West African country, with an estimated population of over 24.65 million [8], has realised some gains in reducing maternal deaths since 1990. Based on current data, the country did not meet its target of a 75% reduction in MMR by the end of 2015. According to the World Health Organization (WHO) report, *Trends in Maternal Mortality: 1990–2013*, MMR declined in Ghana from 760 per 100,000 live births in 1990 to 570 in 2000 and to 380 in 2013 [6], which represents a 50 % reduction in MMR in 23 years. Notwithstanding these advances, the fact remains that Ghana’s progress is less than optimal and much more needs to be done. Ghana was ranked 154 out of 179 countries assessed in the 2015 annual *State of the World’s Mothers* report, which ranks the wellbeing of mothers in various countries. The assessment is based on several indicators. Ghana’s overall ranking was low with a 1 in 66 lifetime risk of maternal death, under-five mortality rate of 78.4 per 1,000 live births, 11.5 expected number of years of formal education, gross national income per capita of US$1,770 and only 10.9 % of government seats were held by women. Additionally, Ghana is one of 11 countries with the greatest urban child survival gaps, that is, poor urban children are 3 to 5 times as likely to die as their rich counterparts [9]. Such inequities are often reflected in poor living conditions, making these mothers and children even more vulnerable to ill-health. Conditions such as poor sanitation, food insecurity, limited access to perinatal care and skilled birth attendance lead to poorer health outcomes. As pointed out in the report, average statistics for health indicators often mask inequalities [9], even when it appears progress is being made on those indicators. Therefore, methods of data collection and analysis require particular consideration, especially disaggregated data, which provides valuable information for healthcare planning.

## Causes of maternal deaths in Ghana

The major contributors to maternal deaths in Ghana are attributable to direct obstetric causes. A retrospective cohort study in one of the major teaching hospitals in Ghana revealed that the top five causes were hypertensive disorders of pregnancy, haemorrhage, genital tract sepsis (including septic abortion-related complications), early pregnancy deaths (including ectopic pregnancy, abortion-related complications and molar pregnancy), and infection, with the first four accounting for about two-thirds of all deaths [10]. Another study based on a a 5-year retrospective survey, reports haemorrhage, abortion, hypertensive disorders of pregnancy, sepsis and obstructed labour as the major causes of maternal deaths. It also lists major infectious diseases, such as malaria and viral hepatitis, as well as non-infectious conditions like anaemia [11]. Similar findings have been reported by other researchers [12, 13].

As a consequence of the slow progress by many countries in reaching MDG targets, the MDG Acceleration Framework was developed in 2010, by the United Nations Development Programme and other United Nations’ agencies. The MDG Acceleration Framework provides a systematic approach for countries not achieving MDG targets to identify possible causes for the delay and find solutions to hasten progress [14]. For this reason, the framework is different for each country and is tailored towards aspects of the Millennium Declaration that a country has been slow at realising. Justifiably, Ghana’s MDG Acceleration Framework Country Action Plan, developed in 2011, focuses on MDG 5, in a bid to intensify efforts to overcome barriers to reducing maternal deaths [15]. Three areas identified as requiring prioritised interventions are family planning, skilled delivery services, and emergency obstetric and neonatal care [14].

Policy guidelines on reproductive healthcare in Ghana include evidence-based best practices adopted to enhance care. For instance, active management of the third stage of labour is recognised as an effective strategy to prevent post-partum bleeding. This strategy was introduced jointly in 2003 by the International Federation of Obstetrics and Gynaecology and the International Confederation of Midwives [16]. Active management of the third stage of labour consists of three components which are intended to facilitate the delivery of the placenta and prevent post-partum bleeding. It was later adopted by Ghana as outlined in the 2008 *National Safe Motherhood Protocol* of the Ghana Health Service [17]. Nonetheless, a recent evaluative study points out that, despite having received training in the use of the protocol, all the components involved are not followed consistently by maternal health personnel when attending to births, due to multiple reasons such as inadequate support from colleagues and high workload [18]. Similarly, a criteria-based audit of quality of care in severe pre-eclampsia and eclampsia indicated a 15–85 % mean adherence to nine key clinical care protocols [19], revealing significant gaps in practice. Other evidence-based guidelines (mostly local adaptation of internationally accepted standards) identified in a desk review [20] include the following:

- The National Family Planning Protocols Manual (2007) [21];
- Reproductive Health Service Policy and Standards (2003) [22];
- Prevention and Management of Unsafe Abortion - Comprehensive Abortion Care Services, Standards and Protocol (2006) [23]; and
- Improving Access to Quality Care in Family Planning, Medical Eligibility Criteria for Contraceptive Use (2008) [24].

Within the national guidelines are strategies for the prevention and treatment of the major causes of maternal deaths. These include use of Oxytocin/Misoprostol in the management of haemorrhage and post-abortion complications; management of eclampsia with Magnesium Sulphate; and prevention of sepsis by using clean delivery kits and antibiotics. Data from studies in resource-poor settings support the use of criteria-based clinical audits as a useful tool for identifying gaps in obstetric practice and providing targeted information on areas requiring quality improvement or intervention [19, 25–27]. Application of such audits could be valuable for monitoring care and guiding improvement plans for increasing adherence to standard protocols in Ghana.

## Discussion

### Major maternal health challenges and initiatives in Ghana

The following discussion is centred on Ghana’s continuing efforts to reduce MMR and includes an evaluation of some of the difficulties encountered. These difficulties include:

- challenges in tracking of maternal deaths;
- midwifery workforce crisis;
- challenges in community-based health services;
- poor transport and emergency response; and
- low skilled attendance at birth.

#### Ghana’s continuing efforts

Multiple challenges have prevented developing countries from making good progress in reducing maternal deaths, including inefficient maternity referral systems [28], inadequate numbers and uneven distribution of skilled health personnel [29] and sociocultural barriers to use of obstetric services [30]. Ghana has so far adopted various strategies in response to specific maternal health challenges. A significant measure introduced in 2008, to improve equity in access to maternity care services is the provision of free maternal healthcare services for all pregnant women through the Ghana National Health Insurance Scheme [31]. Interestingly, family planning services are not covered under the scheme, despite evidence that the country has significant unmet need for family planning. For instance, in 2014, unmet need for family planning was 30 % among married women alone and 42 % among unmarried sexually active women [32]. This unmet need may translate into higher abortion rates with associated abortion-related complications. Given that abortion-related complications are a significant contributor to maternal deaths in the country [10–13], it is unclear why the country’s Ministry of Health has yet to include family planning services in the health insurance benefit package of maternal healthcare [33]. In 2012, changes to the Ghana National Health Insurance Act (Act 852) mandated the health minister to prescribe the healthcare benefits package and include any family planning package under the National Health Insurance Scheme [34]. Despite passage of the law (Act 852), it is yet to be operational [35]. In the longer term, eliminating Ghana’s unmet need for contraception through its inclusion in the National Health Insurance Scheme will offer cost-saving benefits for the country through the reduction of unintended pregnancies, prevention of abortion-related complications and general improvement in maternal health [35]. Nonetheless, despite these deficiencies, some advances have been made in Ghana. For instance, skilled attendance at birth has shown an increase from 40 % in 1988 to 74 % in 2014 [32].

#### Tracking maternal deaths

One particular difficulty in developing countries is measuring and tracking maternal deaths [6, 36, 37] due to the absence of complete and accurate registration systems [6]. In Ghana, MMR measurement problems stem from a poor vital registration system, poor data collection practices in health facilities, possible misclassification of maternal deaths, and difficulties tracking births that do not occur in health facilities. As a consequence of such difficulties, data may be outdated, unreliable or incomparable [37]. For instance, in a 2014 WHO report, Ghana’s 2013 MMR was given as 380 per 100,000 live births, with a possible lower estimate of 210 and a higher estimate of 720 [6], a wide disparity which may hinder the proper planning and meeting of healthcare needs of the population. MMR measurement problems have real implications for decision-makers with regards to setting realistic targets and efficient allocation of resources [36]. Maternal death reviews are an essential means of capturing maternal deaths by helping estimate maternal mortality while providing insight into ways of improving practice and preventing future maternal deaths [6]. Ghana’s Ministry of Health actively promotes clinical and case audit of maternal deaths [38], which will help improve the quality of obstetric care and documentation. In recent times, there has been an increase in published research based on clinical audits of maternal deaths within the Ghana Health Service to help improve health service provision [12, 19, 39, 40].

#### Midwifery workforce crisis

Midwifery personnel are a critical group of maternal healthcare providers. Even though there is evidence of shortage of healthcare personnel globally, there is no universal measure of the shortage [41]. A yardstick often used to plan midwifery workforce is 6 midwives per 1,000 births in a year [42]. The 2011 *State of the World’s Midwifery* report estimated Ghana’s midwife to population ratio to be 5 per 1000 live births [42], which is close to the WHO’s recommendation. While this ratio compares favourably with some developing countries, and almost meets the WHO’s recommended target for midwifery personnel per population, it must be viewed in the context of the country’s particular circumstances. According to the 2014 *State of the World’s Midwifery* report, due to the ageing population of midwives in Ghana, the country is expecting high workforce losses in a the next 10 years, further depleting the midwifery population [43]. Unfortunately, published data on the current age distribution and specific rates of exit from the workforce is not available. Statistics on workforce availability in 2012 showed that, the sexual, reproductive, maternal and newborn healthcare in Ghana was only 30 % of what is required [43]. The 2012 graduates account for nearly a quarter of the 2014 midwifery workforce, a trend, if maintained, that could significantly increase women’s access to midwifery services. Comparatively, the 2011 and 2014 *State of the World’s Midwifery* reports show a general increase in the total numbers of maternal health staff in Ghana. The number of midwifery personnel has increased from 3,780 to 4,185 with an additional 273 nurses who spend 80 % of their working time on maternal and neonatal services [42, 43]. Midwives provide the majority of skilled maternity care services in Ghana and any discussion of maternal health workforce must consider recruitment, retention and equitable distribution of midwives. Factors such as poor remuneration, lack of incentives, inadequate resources and lack of social amenities inhibit the recruitment and retention of midwifery and other health staff in rural areas, where services are most needed [44–46]. Many developing countries have experienced significant losses of skilled health workforce, including midwives, to high-income countries [47]. In 2009, it was reported that over 24 % of nursing and midwifery personnel trained in Ghana were working abroad [48]. In response to the shortage of midwifery personnel, the government of Ghana has upgraded midwifery training schools and introduced direct-entry midwifery training programmes at the diploma level in 2007 [49] and as Bachelor of Science in 2011 [42]. A third entry pathway is the 2-year post-basic midwifery programme, leading to a diploma, targeted at community health nurses and health assistants from underserved communities, which may help increase the number of midwives in remote areas, due to the shorter duration of training [50]. The impact of the country’s investment in expanding the health workforce needs to be measured to guide further action on current and future needs. However, despite the government’s efforts, there are challenges in retaining these key personnel in remote/rural areas and these challenges need to be addressed to improve retention [46, 51].

Historically, the country’s healthcare efforts have been reactionary to emergency or advancing healthcare system problems rather than being proactive in preventing such problems. For example, major increases in training of professional midwifery personnel were not pursued, until their numbers dipped significantly, despite the known effects of economic migration and advancing age of existing personnel. Such reactionary measures create conditions where problems become deep-rooted and use of short-term strategies make them more costly and time-consuming to resolve. Targeted investments need to be consistently made in human resources planning and training for the maternal health sector. Ultimately, a more cost-effective and sustainable approach is to invest in standardised midwifery training which equips personnel with the minimum competencies required to provide emergency obstetric care [43]. Policymakers also need to consider relevant health-worker needs when developing guidelines to improve retention of key personnel, particularly in remote areas [46, 51].

#### Other maternal healthcare workforce

The midwifery workforce in Ghana is supplemented by other categories of health service workers. The 2014 *State of the World’s Midwifery* report indicates that the number of obstetricians and gynaecologists were 549, a great improvement over 64 obstetricians reported in the 2011 report [42, 43]. This category of specialists could make a real difference if, in reality, their services reach those who need them most.

In recent times, Ghana has focused on training increasing numbers of auxiliary and middle level personnel [52], particularly for service in remote/rural areas. Categories of trained auxiliary healthcare workers include community health nurses (certificate) and health care assistants. Since 2003, efforts were intensified to train more community health nurses for Community-based Health and Planning Services (CHPS). CHPS are a type of healthcare facility in Ghana which plays an important role in reproductive healthcare in remote and rural communities. Although it was initially planned for each CHPS zone to have one community health nurse, it is estimated that currently, there are at least two per zone, suggesting an over-production of this category of health workers for the CHPS zones available [53]. The initial emphasis on auxiliary personnel is, however, not without cost to reproductive healthcare as it has implications for the quality of patient care and does not adequately cater for future workforce requirements. It appears to be an attempt to lower the financial cost of training and retain the personnel since they are deemed less likely to seek career advancement [54]; and unlikely to emigrate [55]. In contrast, the WHO’s recommendations emphasise local recruitment and training, as well as access to professional education and support, as a means to retain health workers in remote areas [56]. The existence of different types of health professionals carrying out maternity care tasks creates particular challenges due to lack of standardisation in training and poorly defined roles [57, 58]. The efficiency and cost-effectiveness of recently introduced low- and mid-level healthcare workers providing maternal health services remains to be fully determined. Conscious efforts must be made to ensure that growth in maternity care workforce is driven mainly by higher numbers of professionally prepared midwifery personnel rather than auxiliary personnel, as it has been demonstrated that professional midwifery care is key to reducing MMR [43].

In Ghana, some pregnant women seek care from multiple sources including traditional practitioners, even where there is access to obstetric care [59–62]. Traditional practitioners may include traditional birth attendants (TBAs), diviners, spiritualists and herbalists [60, 61]. The 2008 *Ghana Maternal Health Survey* report shows that, over half (55 %) of all births occurred in the presence of a skilled attendant, while 29 % were assisted by TBAs [63], although the extent of use may vary across the country. Reasons for preference of such services are often socio-cultural in nature [59]. Therefore, improvement in utilisation of skilled care requires an understanding of the culture and values of service users. A recent study revealed that formal healthcare providers were isolated from the culture of the communities they served, as opposed to traditional providers [60]. Presently, the role of TBAs in Ghana is officially restricted to non-birthing support during pregnancy, hence, TBAs no longer receive training and support for maternal birthing services [61]. Despite the lack of support, pregnant women may continue to use them for antenatal and birthing care because of deep-rooted, traditionally held beliefs [59]. Although the activities of traditional practitioners have an impact on maternal health outcomes, limited engagement of these practitioners makes it difficult to measure the nature and extent of their impact [62]. Without a discontinuation or decline in utilisation of their services by community members, marginalising TBAs and other practitioners means that the country misses an opportunity to reach some of its most vulnerable population.

#### Community-based health and planning services

A significant national health policy initiative implemented by Ghana is the Community-based Health and Planning Services system. The initiative was adopted in 1999 by Ghana’s Ministry of Health to increase geographic access to maternal and child health services, particularly in rural and remote communities [64]. The programme relies on collaboration with community leaders and volunteers to mobilise resources and labour to construct a health facility known as a Community Health Compound, consisting of space for a clinic and accommodation for a health care provider. These compounds are manned by Community Health Nurses and Community Health Officers [63]. Upscale of the CHPS initiative has occurred at different rates in the various districts in Ghana. By 2008, almost all districts had some coverage of functioning CHPS, though districts where the pilot projects took place had up to 90 % or more of their population covered while most others had between 10 to 40 % coverage. On the whole, the population covered by functioning CHPS zones across Ghana is low, especially, in the highly populated regions in southern Ghana [65].

In 2010, the geographical delineation for a CHPS zone was changed to correspond with electoral areas as opposed to being based on population size. In 2013, there were 5,487 CHPS zones, out of which 1,189 were reported to be functional [53]. Though the initiative has been largely successful in reducing distances to health facilities, reports indicate an inadequate skill mix of community health officers, thus affecting the quality and scope of their work, particularly in terms of obstetric care services [46, 66]. This situation is problematic because evidence shows that not only is the availability of healthcare workers important [67], so is their ability to identify and respond effectively to obstetric emergencies [68]. The activities of community-based workers are further limited by inadequate supervision and lack of resources [46, 58]. Also, high attrition rates of community health workers have been reported in some communities [58]. Overall, hard-to-reach areas will be better served if expansion of the CHPS programme and health infrastructure progress in tandem with health resource needs such as essential health personnel.

#### Transport and emergency response

Functional maternity referral systems rely on well-organised emergency medical systems. In Ghana, lower level health facilities, such as district hospitals and CHPS compounds, require a supportive network of maternity referral systems to function effectively and efficiently [40]. Efficient referral systems depend on factors such as good roads, appropriate transportation, information and communications technologies [69]. These factors are deficient in many parts of the country [69]. Maternal deaths in Ghana are therefore highest in rural areas where access to emergency obstetric care is also limited by scarce resources, long distances to health facilities, poor transportation and road networks [69]. Strengthening of its emergency response system is therefore of great importance to Ghana.

Development of emergency medical services in Ghana is still at an early stage. The Ghana National Ambulance Service was established in 2004 by the Ministry of Health, to provide pre-hospital emergency medical care, and transport patients to health facilities. Emergency medical training programmes have also been developed for physicians, nurses and middle level personnel [70]. Currently, national ambulance services are free [71] and although services have expanded to cover all regions in the country, they remain confined to the regional capitals and a few districts [70, 71]. Additionally, public awareness of services provided by the National Ambulance Service is low, coupled with the perception that they are unreliable, due to poor response times [70, 71]. In remote areas of the Upper East Region of Ghana, for instance, women referred to a higher level facility for emergency obstetric care may not have access to an ambulance and may use alternative means of transport, delaying their arrival. In the absence of ambulances, common modes of transportation used include taxis, motorbikes, bicycles and donkey carts [28], which may put ill pregnant women at serious risk. This deficit is well recognised and some new initiatives aim to address the problem. For example, under a program called the Ghana Essential Health Intervention Program (GEHIP), a pilot project was launched in 2012 to strengthen the emergency referral system in the Upper-East Region of Ghana, with the supply of 3-wheeled emergency transport vehicles and communication equipment at community levels [72]. These motorcycles have been modified to serve as ambulances (‘Motorking Ambulances’) [73]. Following the pilot, which improved emergency response times [72], it was later upscaled as the Sustainable Emergency Referral Care (SERC) Initiative [73]. The project was developed taking into consideration the particular context of healthcare delivery in the region and the introduction of 3-wheeled motorcycles was a key strategy. A similar project in a rural district of East-Mamprusi in the Northern Region of Ghana has been reported to ease transport-related challenges while remaining cost-effective [74]. Similarly, innovative approaches in solving healthcare problems could be developed in other remote communities in Ghana, with the support and input of community members.

#### Low skilled attendance at birth

Another major reproductive healthcare challenge is low patronage of skilled care at birth. Though women may be aware of the merits of skilled birth attendance in formal maternity care units, they may not always utilise these services. Unlike antenatal care visits which are flexible and amenable to rescheduling, the onset of labour is less so and care during labour can be compounded by health system barriers and the individual’s particular circumstances at the time. It is concerning, however, when the skilled birth attendance rate is very low relative to antenatal care attendance, especially, among vulnerable populations. For instance, between 1993 and 2014, pregnant women who made antenatal care visits at least once increased from 86 to 97 %, while births occurring in a health facility rose from 42 to 73 % [32]. Rural women remain at a disadvantage as the *2014 Ghana Demographic and Health Survey* reports that, in the 5 years preceding the survey, 91 % of births in urban areas were assisted by a skilled provider, compared to 59 % in rural areas and as low as 36 % in the Northern Region of Ghana [32].

Several factors, including sociocultural factors and perceived quality of healthcare, may have contributed to the situation. Studies have also raised concerns about negative attitudes of health staff, poor communication skills, and low standards of care [75–78]. Other reasons for preference for alternative care, such as home birth or use of TBAs, include the liberty to engage in cultural practices/beliefs, such as use of herbal medicines believed to hasten the birthing process; and the freedom to use various birthing positions of choice. Additionally, TBAs are perceived by some as more empathetic and trustworthy than orthodox healthcare professionals [30].

Strategies to increase use of skilled healthcare services at birth must accommodate the challenges of healthcare providers as well as inputs and expectations of service users. Healthcare workers need to provide a more supportive environment by being sensitive and accommodating to diverse socio-cultural perspectives and employing tact in educating clients or managing their needs. Certain non-harmful socio-cultural practices could form part of comprehensive holistic care at home and in health facilities. Finally, during national health policy development processes, especially when adopting broader global strategies, such as the post-2015 sustainable development agenda [79], health service users from different socio-cultural backgrounds may be better served if their inputs are sought and included through local consultative processes.

## Summary

Ghana’s potential success at reducing maternal deaths is hampered by multiple problems, despite many adopted strategies. Critical among the country’s reproductive health issues are health workforce crisis, infrastructural challenges, poor emergency response system, healthcare policy issues and socio-cultural factors. Without doubt, the MDGs have facilitated more targeted and deliberate actions to eliminate inequities in societies. In Ghana, serious thought needs to be given to strategies adopted previously and improvements made, where appropriate. Current evidence suggests that both targets of MDG5 (significant reduction of MMR and universal access to reproductive health) have not been reached by Ghana. While acknowledging that Ghana is confronted with multiple maternal health issues and has taken some remedial actions, it is also recognised that available evidence on maternal health standards and practices, can help reduce MMR, if properly applied. As the country repositions itself to draw on such knowledge and past experiences, it needs to ensure that policies adopted suit its unique country-specific needs. Also, in re-evaluating maternal health efforts, long-term goals need to receive higher emphasis and sustained attention, which is particularly important if the post-2015 target of reducing global MMR to less than 70 per 100,000 live births by 2030, is to be achieved [79]. Ghana can reduce its MMR considerably by ensuring adherence to evidence-based clinical care protocols, strengthening its midwifery workforce and primary health services, improving transport and emergency response systems, and fortifying measures to increase skilled attendance at birth.

## Abbreviations

CHPS: community-based health and planning services
GEHIP: Ghana essential health intervention program
MDG: millennium development goal
MMR: maternal mortality ratio
SERC: sustainable emergency referral care
TBAs: traditional birth attendants
WHO: World Health Organization
